# The comparison between effects of Taichi and conventional exercise on functional mobility and balance in healthy older adults: a systematic literature review and meta-analysis

**DOI:** 10.3389/fpubh.2023.1281144

**Published:** 2023-12-18

**Authors:** Yiting Li, Meng Liu, Kaixiang Zhou, Gengxin Dong, Brad Manor, Dapeng Bao, Junhong Zhou

**Affiliations:** ^1^School of Sport Medicine and Physical Therapy, Beijing Sport University, Beijing, China; ^2^Sports Coaching College, Beijing Sport University, Beijing, China; ^3^College of Physical Education and Health Science, Chongqing Normal University, Chongqing, China; ^4^Hebrew Senior Life Hinda and Arthur Marcus Institute for Aging Research, Harvard Medical School, Boston, MA, United States; ^5^China Institute of Sport and Health Science, Beijing Sport University, Beijing, China

**Keywords:** exercise prescription, protocol design, rehabilitative programs, Taichi, functional mobility, balance, older adults

## Abstract

**Background:**

Taichi is beneficial for functional mobility and balance in older adults. However, such benefits of Taichi when comparing to conventional exercise (CE) are not well understood due to large variance in study protocols and observations.

**Methods:**

We reviewed publications in five databases. Eligible studies that examined the effects of Taichi on the outcomes of functional mobility and balance in healthy older adults as compared to CE were included. Subgroup analyses compared the effects of different types of CE (e.g., single and multiple-type exercise) and different intervention designs (e.g., Taichi types) on those outcomes (Registration number: CRD42022331956).

**Results:**

Twelve studies consisting of 2,901 participants were included. Generally, compared to CE, Taichi induced greater improvements in the performance of Timed-Up-and-Go (SMD = −0.18, [−0.33 to −0.03], *p* = 0.040, I^2^ = 59.57%), 50-foot walking (MD = −1.84 s, [−2.62 to −1.07], *p* < 0.001, I^2^ = 0%), one-leg stance with eyes open (MD = 6.00s, [2.97 to 9.02], *p* < 0.001, I^2^ = 83.19%), one-leg stance with eyes closed (MD = 1.65 s, [1.35 to 1.96], *p* < 0.001, I^2^ = 36.2%), and functional reach (SMD = 0.7, [0.32 to 1.08], *p* < 0.001, I^2^ = 86.79%) tests. Subgroup analyses revealed that Taichi with relatively short duration (<20 weeks), low total time (≤24 h), and/or using Yang-style, can induce significantly greater benefits for functional mobility and balance as compared to CE. Uniquely, Taichi only induced significantly greater improvements in Timed-Up-and-Go compared to single- (SMD = −0.40, [−0.55 to −0.24], *p* < 0.001, I^2^ = 6.14%), but not multiple-type exercise. A significant difference between the effects of Taichi was observed on the performance of one-leg stance with eyes open when compared to CE without balance (MD = 3.63 s, [1.02 to 6.24], *p* = 0.006, I^2^ = 74.93%) and CE with balance (MD = 13.90s, [10.32 to 17.48], *p* < 0.001, I^2^ = 6.1%). No other significant difference was shown between the influences of different CE types on the observations.

**Conclusion:**

Taichi can induce greater improvement in functional mobility and balance in older adults compared to CE in a more efficient fashion, especially compared to single-type CE. Future studies with more rigorous design are needed to confirm the observations here.

## 1 Introduction

The diminished functional mobility and balance in aging oftentimes lead increased risk of falls and poor quality of life in older adults, which are one of the main target for rehabilitative programs in geriatric practice (1, 2). Numerous studies have shown that Taichi is one promising strategy to improve functional mobility and balance in older adults (3, 4). Taichi is a kind of mind–body exercise with low physical load and consisting of social and enjoyable interactions that are particularly appropriate for older adults (5). As compared to conventional exercise (CE), a type of widely used physical exercise that mainly consists of repetitive movement of parts of the body (e.g., resistance training), Taichi includes a series of whole-body movements that are performed simultaneously and continuously, emphasizing multi-joint coordination. Taichi also combines regulated breathing into the movement routine and more focuses on awareness of body alignment and self-control that can particularly help balance control (6, 7).

Still, inconsistent observations and large variance in the study design (e.g., the styles of Taichi) exists across different studies. For example, Day et al. showed that Taichi induced a greater reduction in time to complete Timed-Up-and-Go test (TUG) but shorter time to maintain one-leg stance as compared to stretching (6); while Son et al. showed that compared to the combined exercise of resistance and balance training, 12-weeks Taichi induced greater improvement in the time to maintain one-leg stance with eyes open (OLS-O) but not in TUG time (8). Additionally, most of systematic literature review and meta-analysis on this topic only compared Taichi to blank control or health education (9). Only one meta-analysis compared Taichi and CE by including only three studies (10). The effects of Taichi on functional mobility and balance as compared to CE is still not well characterized.

In this systematic literature review and meta-analysis, we aimed to quantitatively examine the effects of Taichi on functional mobility and balance in healthy older adults as compared to CE. The findings of this work may ultimately help the design of appropriate strategies implementing Taichi for functional mobility and balance in older adults.

## 2 Methods

### 2.1 Design

This systematic literature review and meta-analysis were conducted using Preferred Reporting Items for Systematic Reviews and Meta-Analysis guidelines (11) and registered with PROSPERO (Registration ID: CRD42022331956).

### 2.2 Search strategy and selection criteria

Five electronic databases [PubMed, EBSCO (databases with SPORT-Discus, MEDLINE, APA psycho), Web of Science, Cochrane, Embase] were used to search articles from the inception until November 29th, 2023. The search strategy followed the PICOS principle (Population, Intervention, Comparison, Outcome, and Study design). The following Medical Subject Headings (MeSH) terms and keywords were used for the search strategy: [‘elderly’ or ‘aged’ or ‘older adults’ or ‘senior’ or ‘older people’ or ‘old’] and [‘functional mobility’ or ‘Functional Movement’ or ‘Physical Functional Performance’ or ‘Functional Performance’ or ‘Functional Status’ or ‘Locomotion’] and [‘Tai Ji’ or ‘Tai Chi’ or ‘Taichi’ or ‘Tai-Ji’ or ‘Tai Ji Quan’ or ‘Taijiquan’ or ‘Taiji’] and [‘randomized controlled trial’ or ‘randomized’ or ‘RCT’].

The inclusion criteria were: (1) the mean age of participants was 60 years and older; (2) Taichi was used as intervention; (3) CE was used as the control; (4) the outcomes were related to functional mobility [e.g., TUG time, sit to stand (STS) time] or balance [i.e., OLS-O, one-leg stance with eyes closed (OLS-C), functional reach (FR)].

The exclusion criteria included (1) those consisting of participants with any overt neurological diseases (e.g., severe cognitive impairment, Parkinson’s disease, etc.) or other conditions (e.g., visual impairment, depression, etc.) that seriously affect balance and mobility; (2) repetitive publication; (3) abstracts, systematic review, case report, and register trials report; (4) non-RCT design; (5) not written in English or unable to obtain outcome data.

### 2.3 Data extraction

The data extraction process was conducted by two authors (YL and ML) according to the Cochrane Collaboration Handbook (12). The data were extracted as follows:

- The first author, publication time of the literature, and publishing country/location.
- Average age and sample size of the research subjects.
- Frequency, time, type of exercise, and period of interventions.
- Outcomes: The primary outcome of functional mobility was TUG time, and the secondary outcomes were STS time, and the time to complete 50-foot walking. The primary outcomes of balance performance were the time to keep balance during OLS-O, and the time of OLS-C, and the secondary outcome was the maximum reaching distance of FR task in standing position.
- Key information for risk assessment of bias

For each included study, the mean and the SD of the pre-tests, post-tests and follow-up tests were extracted. If any relevant data were missing, the corresponding author or authors were contacted via email. One study did not report the outcome with Mean ± SD but the difference in mean change between groups, standard error, and 95%CI. So, we used mean difference (MD) and standard error (SE) for the following analysis. Another study reported the Mean_change_ and Mean_pre_ only (6), so we calculated using the following outcomes using these well-established formulas (12):


$${\text{Mean}}_{\text{post}}={\text{Mean}}_{\text{pre}}+{\text{Mean}}_{\text{change}}$$

$$\text{SDpost}=\sqrt{N}\times \left(UCI-LCI\right)÷(2\times \text{tinv}\left(1-0.95,\;\left(NE+NC-2\right)\right)$$

where the variable N represented the overall sample size of the group; UCI and LCI were the upper and lower limit of the confidence interval, respectively; NE was the sample size of the experimental group; and NC represented the sample size of the control group.

### 2.4 Quality assessment

The quality of the included studies was assessed independently by two authors (YL and ML) based on the guidance in the Cochrane Handbook for Systematic Reviews of Interventions (12). The bias risk assessment mainly includes seven criteria: random sequence generation (selection bias), allocation concealment (selection bias), blinding of participants and personnel (performance bias), blinding of outcome assessment (detection bias), incomplete outcome data (attrition bias), selective reporting (reporting bias), and other bias. The quality of the evidence was also assessed independently by two authors (YL and ML) based on the GRADE criteria. Any score on which the two authors disagreed was discussed with the third author (DB or JZ) until a consensus was achieved.

### 2.5 Statistical analysis

RevMan 5.3 software (Cochrane Collaboration, Oxford, United Kingdom) and Stata version 16.0 (Stata Statistical Software, release 16; Stata Corp., College Station, TX, United States) were used for data analysis. Continuous data were analyzed by combining the mean difference (MD) of each study when the outcome was reported using the same measurement units; or the standardized mean difference (SMD) when the outcome was reported using different measurement units. Specifically, the MD was calculated as the mean difference of the outcomes in the intervention group before and after the intervention minus the mean difference of the outcomes in the control group before and after the intervention (13). The SMD was then calculated as the MD divided by the pooled intervention-specific standard deviation. For studies reporting the MD and standard error (SE), we convert the rest studies with mean ± SD into MD and SE for the next analysis. The magnitude of SMD was classified according to the following scale: 0–0.19 represents negligible effect, 0.2–0.49 represents a small effect, 0.5–0.79 represents moderate effect, and 0.8 represents large effect (14). Value of *p*<0.05 was considered statistically significant. The I^2^ statistic was used to assess the extent of heterogeneity (I^2^ = 0–40%, low; I^2^ = 30–60%, moderate; I^2^ = 50–90%, substantial; I^2^ = 75–100%, considerable). If heterogeneity was not significant (I^2^ < 50%), the fixed effect model was adopted. If heterogeneity was significant (I^2^ ≥ 50%), a random-effects model was used. The meta-regression analysis was used to determine if a factor is a source of heterogeneity (15). Specifically, if the value of p obtained from the regression analysis for a factor was <0.05, this factor would be a source of heterogeneity, and subgroup analysis of this factor was then performed. In addition, publication bias was assessed by generating funnel plots and conducting Egger’s test. If a significant asymmetry was detected (Egger’s test *p* < 0.1), we used Trim and Fill method for sensitivity analysis of the results (16).

## 3 Results

### 3.1 Study selection

**Figure 1:**
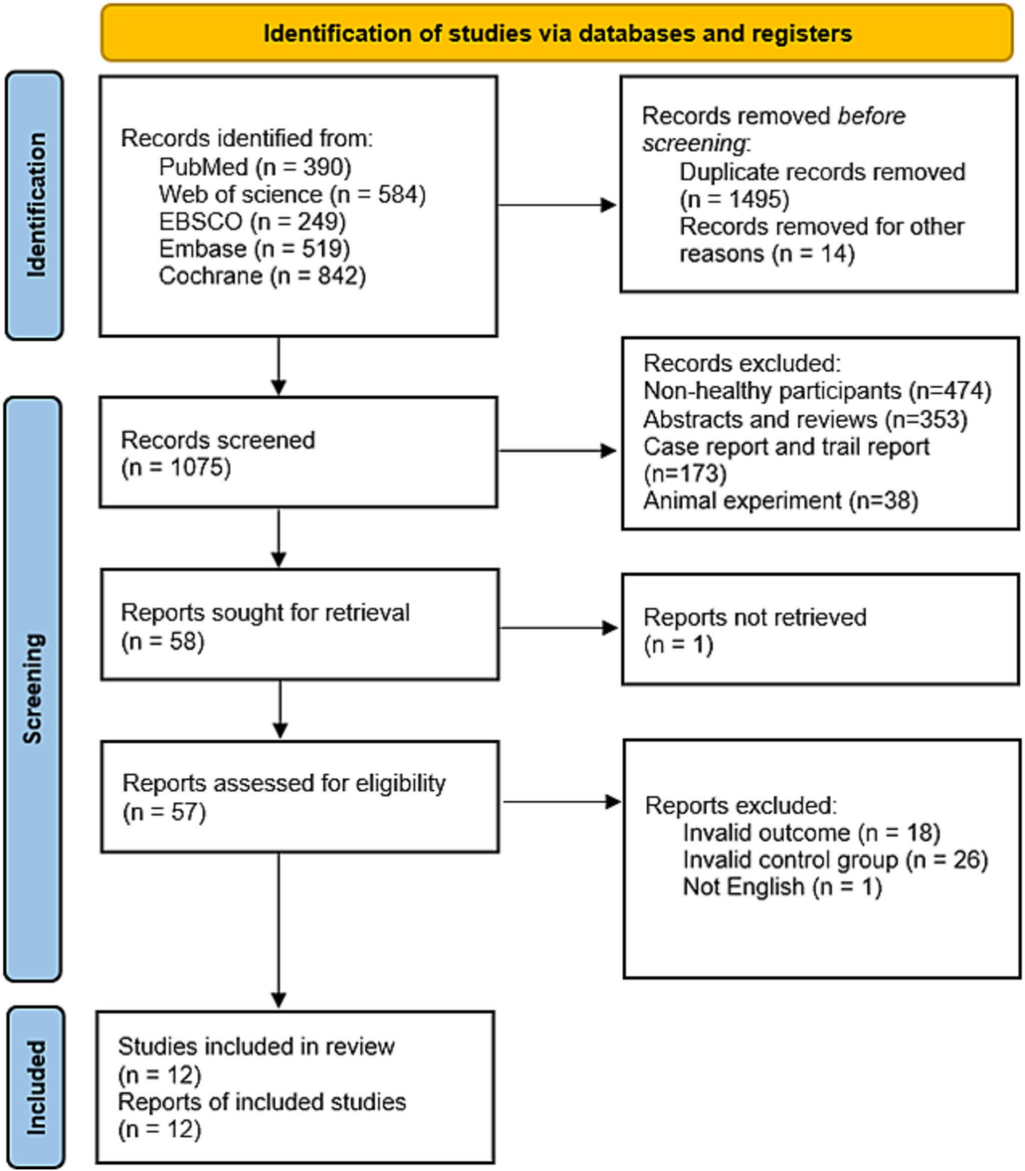
Flow chart for selection of studies.

The flow of the screening process was summarized in Figure 1. The systematic literature search yielded 2,584 records: PubMed (*n* = 390), Web of Science (*n* = 584), EBSCO (*n* = 249), Cochrane (*n* = 842), and Embase (*n* = 519). Among them, we excluded 1,495 repetitive articles, 14 retracted articles, 474 articles consisting of non-healthy participants, 353 abstracts and reviews, 173 case reports and trial reports, and 38 articles of animal experiment. Then 57 articles were excluded by reviewing the full text; Finally, a total of 12 publications were included in this work.

### 3.2 Quality assessment

Six studies used double-blinded design; three used single-blinded; and other three did not report related information. The quality of evidence for functional mobility was moderate; and the quality of evidence for balance was low to moderate (Supplementary Figures S1, S2; Table 1).

**Table 1:** The quality of the evidence (GRADE).

No of studies	Design	Risk of bias	Inconsistency	Indirectness	Imprecision	Other considerations	Absolute effect (95% CI)	Quality	Importance
Timed up and go									
10	randomized trials	serious^1^	no serious inconsistency^2^	no serious indirectness	no serious imprecision	none	SMD -0.18 lower (−0.33 to −0.03 lower)	⊕ ⊕ ⊕Ο MODERATE	CRITICAL
Sit to stand									
4	randomized trials	serious^1^	no serious inconsistency^2^	no serious indirectness	no serious imprecision	none	SMD -0.26 lower (−0.59 to 0.07 lower)	⊕ ⊕ ⊕Ο MODERATE	IMPORTANT
50-foot walking test									
2	randomized trials	serious^1^	no serious inconsistency	no serious indirectness	no serious imprecision	none	MD -1.84 lower (−2.62 to −1.07 lower)	⊕ ⊕ ⊕Ο MODERATE	IMPORTANT
One-leg stance with eyes open									
14	randomized trials	serious^1^	serious^3^	no serious indirectness	no serious imprecision	none	MD 6.00 higher (2.97 to 9.02 higher)	⊕ ⊕ ΟΟ LOW	CRITICAL
One-leg stance with eyes closed									
6	randomized trials	serious^1^	no serious inconsistency	no serious indirectness	no serious imprecision	none	MD 1.65 higher (1.35 to 1.96 higher)	⊕ ⊕ ⊕Ο MODERATE	CRITICAL
Functional reach									
7	randomized trials	serious^1^	serious^3^	no serious indirectness	no serious imprecision	none	SMD 0.7 higher (0.32 to 1.08 higher)	⊕ ⊕ ΟΟ LOW	IMPORTANT

^1^Half of studies did not execute allocation concealment and outcomes measurement blinding. ^2^ Although substantial overall heterogeneity reported, this was downgraded when considering regular exercise type. ^3^ Substantial heterogeneity (I^2^ = 50–90%).

### 3.3 Characteristics of included studies

#### 3.3.1 Participants

A total of 2,901 (Taichi group = 1,446, CE group = 1,455) participants were enrolled in these studies, ranging from 11 to 234 (Table 2). The participants in eight studies were recruited from the local community; four studies did not provide the related information (17, 21, 24, 25). Participants in five studies were with history of falls within 1 year (6, 7, 18, 19, 22); one study recruited non-fallers (21); and six studies did not provide related information (8, 17, 20, 23–25). Six studies recruited inactive (defined as not being involved in any moderate or strenuous activity in the previous 3 months) participants (6, 7, 17, 18, 23, 24), and the other studies did not provide information about it. Participants in five studies had no experience in Taichi and balance training (8, 19, 20, 22, 25), and no such criterion was applied in other studies.

**Table 2:** The extracted study and participant characteristics.

References	Years	Country/location	Sample size (male/female)	Age (years) Mean ± SD	BMI (kg/m^2^)	Weight (kg)
Day et al. (6)	2012	Australia	TC 77/157 CE 64/163	**—**	TC 27.45 ± 3.95 CE 28.06 ± 4.85	**—**
Li et al. (7)	2004	United States	TC 10/52 CE 12/44	TC 75.30 ± 7.8 CE 75.45 ± 7.8	TC 28.01 ± 5.0 CE 28.18 ± 6.4	**—**
Li et al. (17)	2005	United States	TC 38/87 CE 39/92	TC 76.94 ± 4.69 CE 77.99 ± 5.14	**—**	**—**
Li et al. (18)	2018	United States	TC 78/146 CE 80/143	TC 77.5 ± 5.6 CE 77.8 ± 5.3	TC 29.2 ± 6.0 CE 29.4 ± 6.6	**—**
Li et al. (18)	2018	United States	TC 78/146 CE 76/147	TC 77.5 ± 5.6 CE 77.8 ± 5.9	TC 29.2 ± 6.0 CE 29.4 ± 6.6	**—**
Ni et al. (19)	2014	United States	TC 2/9 CE 2/13	TC 70.27 ± 5.69 CE 77.80 ± 7.78	**—**	TC 76.80 ± 22.37 CE 65.84 ± 13.78
Pluchino et al. (20)	2012	United States	TC 8/6 CE 5/9	TC 69.28 ± 6.03 CE 76.00 ± 7.74	**—**	TC 75.81 ± 12.99 CE 73.50 ± 20.57
Son et al. (8)	2016	South Korea	TC 0/26 CE 0/24	TC 72.8 ± 4.7 CE 71.5 ± 3.6	**—**	TC 55.2 ± 7.9 CE 60.7 ± 8.6
Sun et al. (21)	2018	China	TC 0/12 CE 0/13	TC 64.12 ± 3.21 CE 63.26 ± 2.20	TC 25.12 ± 3.19 CE 24.69 ± 2.97	**—**
Taylor et al. (22)	2012	New Zealand	TC 72/161 CE 55/176	TC 75.3 ± 7.0 CE 73.7 ± 6.2	**—**	**—**
Taylor et al. (22)	2012	New Zealand	TC 55/165 CE 55/176	TC 74.4 ± 6.2 CE 73.7 ± 6.2	**—**	**—**
Frye et al. (23)	2007	United States	TC 23 CE 28	69.2 ± 9.26	28.96 ± 4.31	**—**
Audette et al. (24)	2006	United States	TC 0/11 CE 0/8	TC 71.5 ± 4.6 CE 71.3 ± 4.4	**—**	**—**
Yıldırım et al., (25)	2015	Turkey	TC 3/27 CE 4/26	TC 62.9 ± 6.5 CE 64.4 ± 7.5	TC 27.3 ± 4.6 CE 27.5 ± 3.5	**—**

BMI, body mass index; CE, regular exercise; TC, Tai Chi; W, weigh.

#### 3.3.2 Intervention characteristics

The information on the intervention characteristics was presented in Table 3. For Taichi style, four used Sun style, each of 46 (6), 12 (20), 21 (8), and 10 (22) forms; and seven used Yang style of 8 forms in two (7, 18) 10 in two (23, 24), 12 in one (25), and 24 in another two studies (17, 21). Only one study used the modified 18-form Chen style (19).

**Table 3:** Characteristics of the interventional protocol.

References	Groups	Interventions	Frequency (days/week)	Duration (weeks)	Total time (hours)	Outcome
Day et al. (6)	TC	46-form Sun style	2	24	48	TUG↑ STS↑ OLS-O↑
	CE	Stretching	2	24	48	
Li et al. (7)	TC	8-form Yang style	3	24	72	STS↑ 50-foot walking↑ OLS-O↑
	CE	Stretching	3	24	72	
Li et al. (17)	TC	24-form Yang style	3	24	72	TUG↑ 50-foot walking↑ OLS-O↑ OLS-C↑ FR↑
	CE	Stretching	3	24	72	
Li et al. (18)	TC	8-form Yang style	2	24	48	TUG→ STS→ FR→
	CE	Aerobic, resistance, balance and stretching exercise	2	24	48	
Li et al. (18)	TC	8-form Yang style	2	24	48	TUG↑ STS↑ FR↑
	CE	Stretching	2	24	48	
Ni et al. (19)	TC	18-form Chen style	2	12	24	TUG↑ OLS-O↑ FR↑
	CE	Balance training	2	12	24	
Pluchino et al. (20)	TC	12-form Sun style	2	8	16	TUG→ OLS-O→ FR→
	CE	Balance training	2	8	16	
Son et al. (8)	TC	21-form Sun style	2	12	24	TUG↓ STS↓ OLS-O↑ FR↓
	CE	Resistance training, balance training	2	12	24	
Sun et al. (21)	TC	24-form Yang style	5	16	80	OLS-O→ OLS-C→
	CE	Aerobic exercise	5	16	80	
Taylor et al. (22)	TC	10-form Sun style	1	20	20	TUG→
	CE	Aerobic exercise, resistance training, stretching exercise	1	20	20	
Taylor et al. (22)	TC	10-form Sun style	2	20	40	TUG→
	CE	Aerobic exercise, resistance training, stretching exercise	2	20	40	
Frye et al. (23)	TC	10-form Yang style	3	12	36	TUG→
	CE	Aerobic, resistance, balance and stretching exercise	3	12	36	
Audette et al. (24)	TC	10-form Yang style	3	12	36	OLS-O↑ OLS-C↑
	CE	Aerobic exercise	3	12	36	
Sun et al. (25)	TC	12-form Yang style	3	12	36	OLS-O↑
	CE	Aerobic exercise, resistance training, stretching	3	12	36	

TC, Taichi; CE conventional exercise; FR, functional reach test; OLS-O, one-leg stance with eyes open; OLS-C, one-leg stance with eyes closed; STS, sit to stand; TUG, Timed Up and Go. ↑Indicates that the statistically significant change is an increase in the performance outcome. →Indicates that there is no statistically significant change in the performance outcome. ↓Indicates that the statistically significant change is a decrease in the performance outcome. Where possible, a specific value of p was included for each study.

All studies used 60-min sessions, including warm-up, Taichi performance, and relaxation. The weekly frequency was designed as twice a week in six studies (6, 8, 18–20, 22), three times (7, 23–25) in four, five times (21) in one, and once (22) in another one. The intervention period ranged from 8 to 24 weeks, including one study with 8 weeks (20), five with 12 weeks (8, 19, 23–25), one with 16 weeks (21), one with 20 weeks (22), and four with 24 weeks (6, 7, 17, 18).

In terms of the design of CE, seven studies used single-type CE, that is, three (6, 7, 18) used stretching exercise; two (19, 20) used balance training (e.g., maintaining balance on compliant surfaces); two (21, 24) used brisk walking, and five (8, 18, 22, 23, 25) used component exercise (i.e., exercises that combined two or more types of CE).

### 3.4 Study outcomes

Functional mobility was assessed by using TUG time in 11 studies (6, 8, 17–20, 22, 23, 25), STS time in five (6–8, 18), and 50-foot walking time in two (7, 17).

To note, walking distances of TUG were different. Specifically, 3 meters were used in five studies (8, 20, 22, 25), 7 meters in two (18), and 8 feet in another two (19, 23). In STS test, time taken to stand up and sit down was measured with different numbers of repetitions such as 1 time (18), 3 times (6), and 5 times (7, 8).

Balance was assessed by using OLS-O time in 14 studies (6–8, 17, 19–21, 24, 25), OLS-C time in six studies (17, 21, 24, 25), and FR distance in seven (8, 17, 19–21).

#### 3.4.1 Short-term assessments

Two studies completed the post-intervention assessments within 1 week following the last intervention session (19, 20); one completed within 2 weeks (23); and the other completed the assessments immediately after intervention (7, 8, 17, 18, 21, 22, 24, 25).

#### 3.4.2 Long-term assessments

TUG time was assessed at the 6th and 12th month (22) after the intervention in one study (22) and at 6th month in another (21). One study assessed OLS-O time and OLS-C time after 1 and 2 months following the intervention (21), and another study assessed them after 6 months (17).

#### 3.4.3 Effects of Taichi on functional mobility and balance

For functional mobility, five studies showed that compared to CE, Taichi could improve functional mobility [i.e., improvement of TUG time in four studies (6, 17–19), that of STS time in three (7, 18, 19) and 50-foot walking time in two (7, 17)]. For balance, seven studies showed that compared to CE, Taichi could improve balance [i.e., improvement of OLS-O time in six studies (6–8, 17, 19–21, 24, 25), that of OLS-C time in four (17, 21, 24, 25) and FR distance in five (8, 17–20)].

### 3.5 Meta-analysis

All 12 studies were included in the meta-analysis. Uniquely, one study (6) did not report Mean_post_ and SD_post_ of TUG and STS, which was thus not included in the analysis for these two outcomes. Because TUG [e.g., 3 m (8, 17, 20, 22), 7 m (18), 8 foot (19, 23)] and STS [e.g., one-time sit-to-stand (18), five-time repeated sit-to-stand (7, 8)] performance was measured in different protocols, and FR distance was measured in different units [e.g., inch (17, 18), cm (8, 19, 20)] across the included studies, we used SMD of the performance for them. For the performance of 50-foot walking time, OLS-O and OLS-C, we used MD.

Based on the information from the systematic literature review as provided above, we specifically assessed the short-term and longer-term effects of Taichi on functional mobility and balance as compared to CE, and examined the potential impacts from Taichi styles (i.e., Sun style, Yang style), CE types [i.e., single-type exercise (*n* = 8) or multiple-type exercise (n = 6), and with balance (*n* = 5) or not (*n* = 9)], and weekly frequency (≤2 or > 2 times/week), total duration (i.e., <20 weeks or ≥ 20 weeks), and time (≤24 h or > 24 h) of intervention (Table 4).

**Table 4:** Overall and subgroup analysis results regarding the effects of TC compared to CE.

Outcomes	Overall and subgroup analysis	Number of studies	Meta-reg	MD or SMD (95% CI)	Value of *p*	Test of heterogeneity		
						χ^2^	Value of *p*	I^2^ (%)
TUG	Overall	10		SMD -0.18 (−0.33, −0.03)	0.04	22.26	0.01	59.57
	CE type		0.004					
	Single-type	4		SMD -0.40 (−0.55, −0.24)	<0.001	3.2	0.36	6.14
	Multiple-type	6		SMD -0.02 (−0.13, 0.07)	0.54	2.81	0.73	<0.01
	CE with balance		0.49					
	No	5		SMD -0.22 (−0.40, −0.04)	0.02	11.66	0.02	65.71
	Yes	5		SMD -0.12 (−0.45, 0.20)	0.49	8.06	0.09	50.37
	Style		0.12					
	Sun style	4		SMD -0.05 (−0.17, 0.07)	0.44	0.73	0.87	0
	Yang style	5		SMD -0.21 (−0.44, 0.02)	0.07	12.86	0.01	68.89
	Chen style	1		SMD -1.09 (−1.93, −0.26)				
	Frequency (times/week)		0.52					
	≤2	7		SMD -0.14 (−0.30, 0.02)	0.08	13.72	0.03	56.28
	>2	3		SMD -0.23 (−0.65, 0.18)	0.27	5.17	0.08	61.3
	Duration (weeks)		0.79					
	<20	5		SMD -0.25 (−0.66, 0.16)	0.23	8.17	0.09	51.04
	≥20	5		SMD -0.16 (−0.33, −0.00)	0.05	13.96	0.01	71.35
TUG	Total time (hours)	0.97						
	≤24	4		SMD -0.24 (−0.62, 0.15)	0.24	6.53	0.09	54.09
	>24	6		SMD -0.18 (−0.36, 0.00)	0.06	14.89	0.01	66.42
TUG follow-up	After 6 months	3		SMD -0.15 (−0.32, 0.02)	0.08	4.05	0.13	50.59
	After one year	2		SMD -0.18 (−0.31, −0.05)	0.01	0.24	0.62	0
STS	Overall	4		SMD -0.26 (−0.59, 0.07)	0.13	17.51	<0.001	82.87
	CE type	0.06						
	Single-type	2		SMD -0.54 (−0.71, −0.37)	<0.001	0.49	0.48	0
	Multiple-type	2		SMD -0.04 (−0.21, 0.14)	0.68	0.5	0.48	0
	CE with balance	0.06						
	No	2		SMD -0.54 (−0.71, −0.37)	<0.001	0.49	0.48	0
	Yes	2		SMD -0.04 (−0.21, 0.14)	0.68	0.5	0.48	0
OLS-O	Overall	14		MD 6.00 (2.97, 9.02)	<0.001	77.32	<0.001	83.19
	CE type	0.72						
	Single-type	12		MD 6.22 (2.94, 9.50)	<0.001	77.22	<0.001	85.76
	Multiple-type	2		MD 4.52 (−1.91, 10.96)	0.17	0.06	0.81	0
OLS-O	CE with balance	0.003						
	No	10		MD 3.63 (1.02, 6.24)	0.006	35.89	<0.001	74.93
	Yes	4		MD 13.90 (10.32, 17.48)	<0.001	3.19	0.36	6.1
	Style	<0.001						
	Sun style	4		MD 0.29 (−3.43, 4.02)	0.88	5.79	0.12	48.21
	Yang style	8		MD 5.03 (3.57, 6.50)	<0.001	7.3	0.4	4.05
	Chen style	2		MD 14.79 (11.19, 18.38)	<0.001	0.1	0.75	0
	Frequency (times/week)	0.84						
	≤2	8		MD 5.91 (1.28, 10.55)	0.01	64.65	<0.001	89.17
	>2	6		MD 6.02 (4.22, 7.81)	<0.001	4.31	0.51	0
	Duration (weeks)	0.01						
	<20	8		MD 10.28 (5.93, 14.64)	<0.001	14.06	0.05	50.22
	≥20	6		MD 2.89 (−0.00, 5.77)	0.05	29.43	<0.001	83.01
	Total time (hours)	0.003						
	≤24	4		MD 13.9 (10.32, 17.48)	<0.001	3.19	0.36	6.1
	>24	10		MD 3.63 (1.02, 6.24)	0.006	35.89	<0.001	74.93
OLS-O Follow-up	After 2 months	4		MD 5.14 (2.10, 8.18)	<0.001	5.44	0.14	44.88
	After 6 months	2		MD 6.45 (4.59, 8.31)	<0.001	0.43	0.51	0
OLS-C	Overall	6		MD 1.65 (1.35, 1.96)	<0.001	7.84	0.17	36.2
	Duration (weeks)	0.67						
	<20	4		MD 2.16 (0.57, 3.74)	0.008	6.86	0.08	56.25
	≥20	2		MD 1.63 (1.33, 1.94)	<0.001	0.23	0.45	0
FR	Overall	7		SMD 0.7 (0.32, 1.08)	<0.001	45.41	<0.001	86.79
	CE type	0.28						
	Single-type	5		SMD 0.92 (0.56, 1.29)	<0.001	13.03	0.01	69.03
	Multiple-type	2		SMD 0.11 (−0.06, 0.29)	0.21	0.18	0.67	0
	CE with balance	0.97						
	No	2		SMD 0.74 (0.58, 0.89)	<0.001	0.21	0.65	0
	Yes	5		SMD 0.77 (0.11, 1.44)	0.02	25.88	<0.001	84.54
	Style	0.73						
	Sun style	2		SMD 0.25 (−0.20, 0.69)	0.28	0.01	0.91	0
	Yang style	3		SMD 0.53 (0.08, 0.97)	0.02	27.18	<0.001	92.64
	Chen style	2		SMD 1.90 (1.23, 2.57)	<0.001	0.05	0.83	0
FR	Frequency (times/week)							
	≤2	6						
	>2	1		SMD 0.71 (0.25, 1.16)	<0.001	39.65	<0.001	87.39
	Duration (weeks)		0.48					
	<20	4		SMD 1.02 (0.11, 1.93)	<0.001	16.31	<0.001	81.61
	≥20	3		SMD 0.53 (0.08, 0.97)	<0.001	27.18	<0.001	92.64
	Total time (hours)		0.48					
	≤24	4		SMD 1.02 (0.11, 1.93)	<0.001	16.31	<0.001	81.61
	>24	3		SMD 0.53 (0.08, 0.97)	<0.001	27.18	<0.001	92.64

TUG, Timed Up and Go; STS, sit to stand; OLS-O, one-leg stance with eyes open; OLS-C, one-leg stance with eyes closed; FR, functional reach test; MD, mean difference; SMD, standard mean difference.

#### 3.5.1 Short-term effects of Taichi on functional mobility

##### 3.5.1.1 Effects of Taichi on TUG

**Figure 2:**
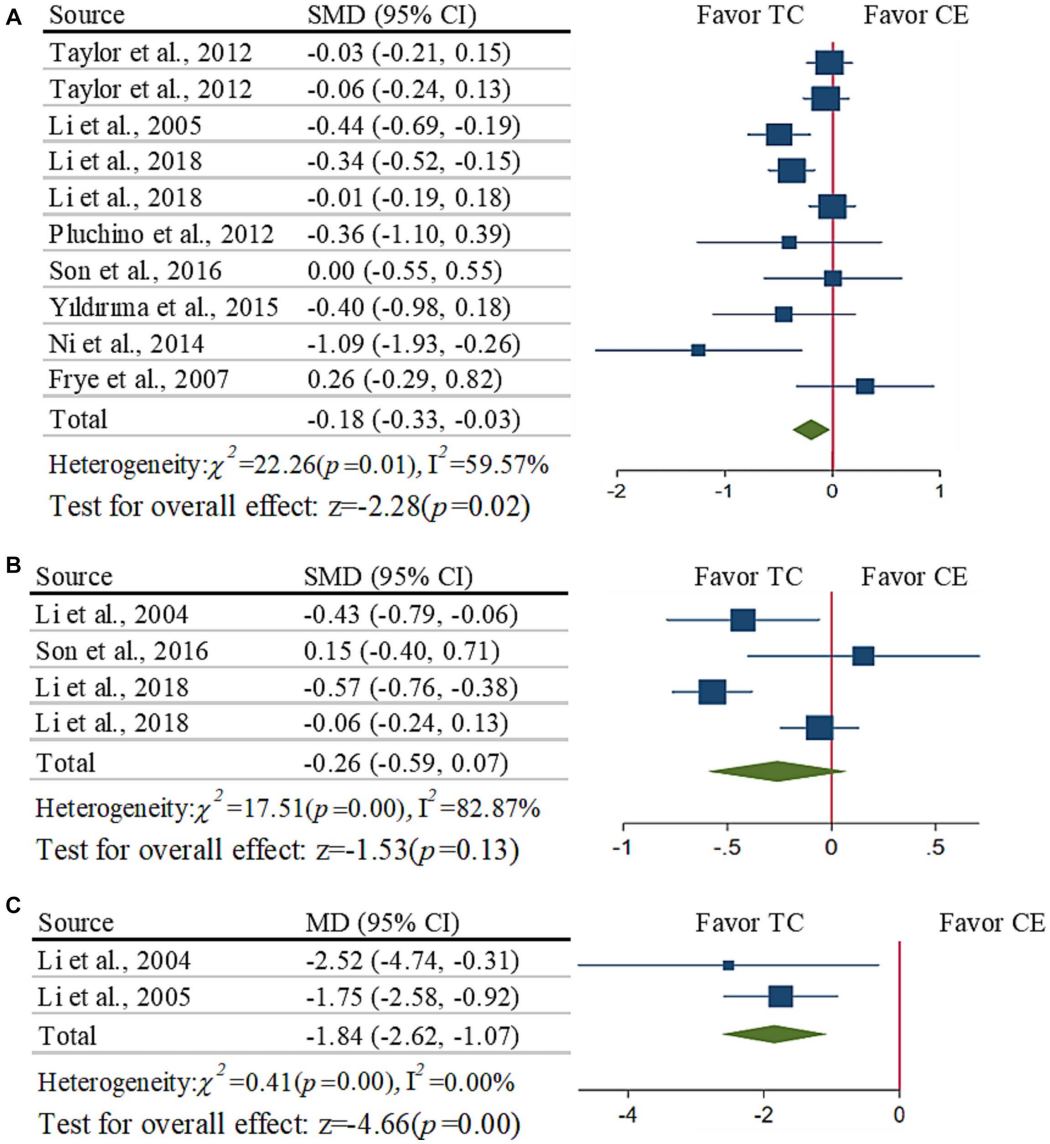
Meta-analysis of the effects of functional mobility: **(A)** TUG time; **(B)** STS time; **(C)** 50-foot walking time.

As compared to CE, Taichi induced greater improvement in TUG time (Figure 2A, SMD = −0.18, [−0.33 to −0.03], *p* = 0.040, I^2^ = 59.57%). The funnel plot and Egger’s test (*t* = −0.82, *p* = 0.44) indicated no publication bias. Sensitivity analysis showed three studies had a much larger effect size than the other (17–19). The pooled effect size was changed (SMD = −0.04, [−0.14 to 0.06], *p* = 0.470, I^2^ = 0%) after removing these three studies.

The subgroup analysis demonstrated that Taichi induced significantly greater reduction in TUG time compared to single-type (SMD = −0.40, [−0.55 to −0.24], *p* < 0.001, I^2^ = 6.14%), which was not significant when compared to multiple-type (SMD = −0.02, [−0.13 to 0.07], *p* = 0.540, I^2^ = 0%). No significant impacts were induced by other aspects (i.e., CE with balance or not, Taichi style, weekly frequency and total time of intervention; *p* > 0.120).

##### 3.5.1.2 Effects of Taichi on STS

As compared to CE, Taichi did not induce significantly greater improvement (Figure 2B, SMD = −0.26, [−0.59 to 0.07], *p* = 0.130, I^2^ = 82.87%). The funnel plots (*t* = 0.31, *p* = 0.790) are symmetrical. Sensitivity analysis showed two studies had a much larger effect size than the other studies (7, 18). The pooled effect size was changed (SMD = −0.04, [−0.21 to 0.14], *p* = 0.680, I^2^ = 0%) after removing these two studies.

The subgroup analysis demonstrated that Taichi induced significantly greater reduction in STS time as compared to CE of single-type (SMD = −0.54, [−0.71 to −0.37], *p* < 0.001, I^2^ = 0%) and/or with balance training (SMD = −0.54, [−0.71 to −0.37], *p* < 0.001, I^2^ = 0%), which was not significant when compared to CE of multiple-type (SMD = −0.04, [−0.21 to −0.14], *p* = 0.680, I^2^ = 0%) and/or without balance training (SMD = −0.04, [−0.21 to −0.14], *p* = 0.680, I^2^ = 0%).

##### 3.5.1.3 Effects of Taichi on 50-foot walking

Taichi induced significant improvement in 50-foot walking time (Figure 2C, MD = −1.84 s, [−2.62 to −1.07], *p* < 0.001, I^2^ = 0%), and the funnel plot is symmetrical. Only two studies were here, we did not perform the subgroup analysis.

#### 3.5.2 Long-term effects of Taichi on functional mobility

The benefits of Taichi for TUG time can sustain after 6 months (SMD = −0.16, [−0.25 to −0.07], *p* < 0.001, I^2^ = 13.09%) and 12 months (SMD = −0.18, [−0.31 to −0.05], *p* = 0.010, I^2^ = 0%). We did not perform analysis on STS and 50-foot walking due to lack of data.

#### 3.5.3 Short-term effects of Taichi on balance

##### 3.5.3.1 Effects of Taichi on OLS-O

**Figure 3:**
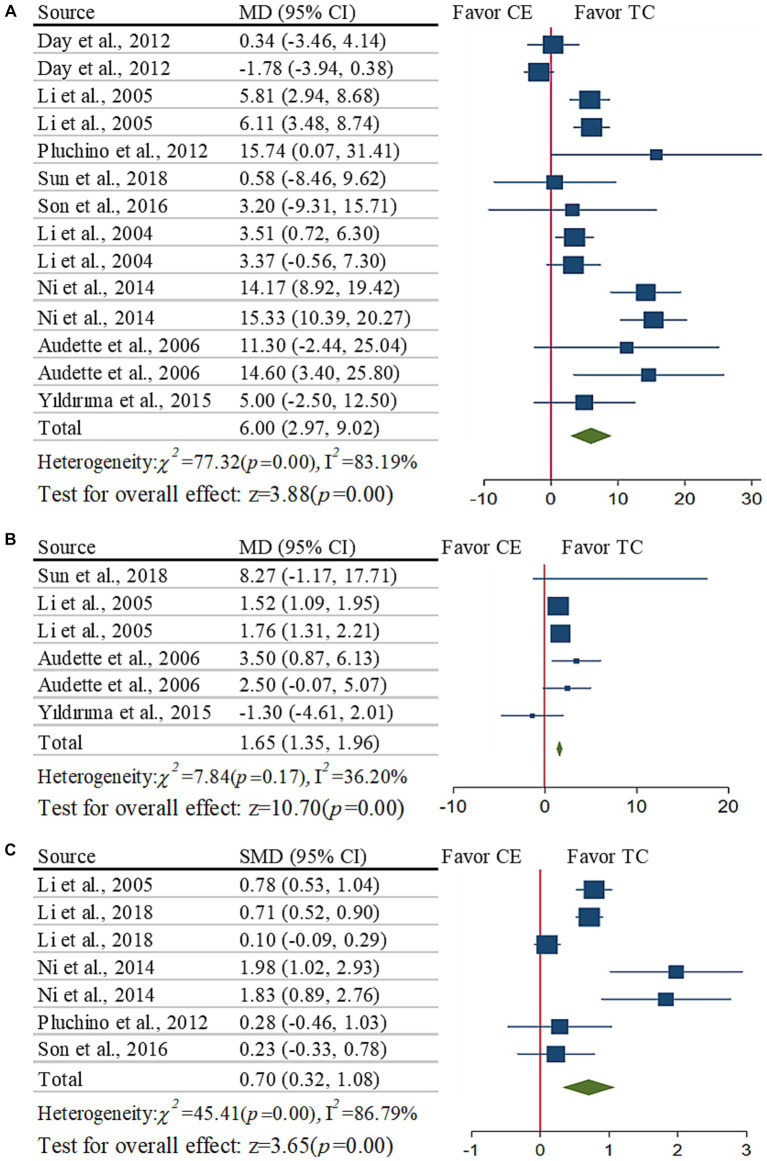
Meta-analysis of the effects of balance: **(A)** OLS-O time; **(B)** OLS-C time; **(C)** FR distance.

Taichi induced significant improvement in OLS-O time (Figure 3A, MD = 6.00s, [2.97 to 9.02], *p* < 0.001, I^2^ = 83.19%) as compared to CE. The funnel plot and Egger’s test (*t* = 1.80, *p* = 0.10) indicated no publication bias. We removed four studies that had a larger effect size than the other studies following sensitivity analysis (6, 19), and the I^2^ dropped from 83.19 to 2% with no change occurring (MD = 5.10s, [3.67 to 6.53], *p* < 0.001, I^2^ = 2%).

The subgroup analysis showed that Taichi induced significantly greater improvement in OLS-O as compared to CE both with (MD = 13.90s, [10.32 to 17.48], *p* < 0.001, I^2^ = 6.1%) or without (MD = 3.63 s, [1.02 to 6.24], *p* = 0.006, I^2^ = 74.93%) balance training, with significant difference between the two subgroups (*p* = 0.003). Taichi style contributed significantly to such benefits (*p* < 0.001), that is, Yang style (MD = 5.03 s, [3.57 to 6.50], *p* < 0.001, I^2^ = 4.05%) had greater benefits than Sun style (MD = 0.29 s, [3.43 to 4.02], *p* = 0.880, I^2^ = 48.21%). Within shorter duration time (<20 weeks, MD = 10.28 s, [5.93 to 14.64], *p* < 0.001, I^2^ = 50.22%) and/or less total time (≤24 h, MD = 13.9 s, [10.32 to 17.48], *p* < 0.001, I^2^ = 6.1%), but no longer duration (≥20 weeks, MD = 2.89 s, [−0.00 to 5.77], *p* = 0.050, I^2^ = 83.01%) and/or more total time (>24 h, MD = 3.63 s, [1.02 to 6.24], *p* = 0.006, I^2^ = 74.93%), Taichi can induce greater improvements as compared to CE.

##### 3.5.3.2 Effects of Taichi on OLS-C

Taichi induced significantly greater improvement (Figure 3B, MD = 1.65 s, [1.35 to 1.96], *p* < 0.001, I^2^ = 36.2%) in OLS-C time as compared to CE. The funnel plot and Egger’s test (*t* = 0.66, *p* = 0.55) indicated that no publication bias.

The subgroup analysis showed that within shorter duration time (<20 weeks, MD = 2.16 s, [0.57 to 3.74], *p* = 0.008, I^2^ = 56.25%), but not in longer duration (≥20 weeks, MD = 1.63 s, [1.33 to 1.94], *p* < 0.001, I^2^ = 0%), Taichi can induce significantly greater improvement as compared to CE. Due to the lack of enough studies, we did not perform other subgroup analysis.

##### 3.5.3.3 Effects of Taichi on FR

The random-effects model showed that Taichi induced significantly greater improvement in FR distance (Figure 3C, SMD = 0.7, [0.32 to 1.08], *p* < 0.001, I^2^ = 86.79%) compared to CE. The funnel plot and Egger’s test (*t* = 1.08, *p* = 0.33) indicated no publication bias. We removed three studies that had larger effect size than the other studies (18, 19) following sensitivity analysis, and the I^2^ dropped from 86.79 to 32% with no change occurring (SMD = 0.65, [0.44 to 0.85], *p* < 0.001, I^2^ = 32%). The subgroup analysis demonstrated no significant impact from any of those aspects we analyzed (*p* > 0.28).

#### 3.5.4 Long-term effects of Taichi on balance

It was observed that the benefits of Taichi for OLS-O (MD = 6.45 s, [4.59 to 8.21], *p* < 0.001, I^2^ = 0%) and OLS-C time (MD = 1.65 s, [1.35 to 1.95], *p* < 0.001, I^2^ = 0%) can sustain after 6 months. We did not perform analysis on FR distance due to lack of data.

## 4 Discussion

This is the first systematic literature review and meta-analysis demonstrating that Taichi is a more efficient strategy to improve functional mobility and balance in relatively healthy older adults as compared to CE. Subgroup analyses further revealed that when the intervention length was short (<20 weeks) and/or the total time was low (≤24 h), Taichi, especially Yang style Taichi, can induce significantly greater benefits for functional mobility and balance; and the types of CE may contribute the observations. The knowledge from this work suggests that Taichi should be carefully considered in future studies and routines of rehabilitative programs for balance and mobility in older adults.

Both TUG and 50-foot walking tests require the capacity of coordinating complex motions of the lower extremities, weight shifting, and dynamic balance, as well as the lower body strength and agility (26). Compared to CE, especially single-type CE (only comparable effects of Taichi to multiple-type CE were observed), Taichi consists of a series of complex whole-body movements and emphasizes weight shifting and hip-knee-ankle coordination (7), thus inducing significant benefits for the performance of TUG and 50-foot walking. On the other hand, the performance of STS is dependent mainly upon the lower-limb muscle strength, which may thus be augmented to similar extent by both Taichi and CE. Therefore, compared to CE, Taichi induced significantly faster TUG time and better 50-foot walking performance, but not by STS time, when comparing to single-type.

It is known to all that the integration of sensory inputs, including vision, proprioception and vestibular sensation, is critical to maintain standing balance, and the “weight” of different types of sensation (i.e., relative contribution) in the regulation of standing balance is changing between scenarios. For example, as compared to OLS-O, the proprioception and vestibular sensation are dominant in OLS-C since the vision is cut off in this condition. Though Taichi was believed as one type of balance training previously (27), we here observed that Taichi can induce significantly greater improvements in OLS-O and FR as compared to regular type balance training, indicating the unique benefits of Taichi for balance control. This is consistent with previous meta-analysis (9). Studies have shown that Taichi can simultaneously augment the sensory perception, facilitate appropriate sensory reweighting process (28, 29), and reduce the reaction time of lower-extremity muscles (e.g., tibialis anterior), which may contribute to the Taichi-induced improvements in balance control we observed here.

Meanwhile, the cognitive function (e.g., attention, executive function) have been closely linked to mobility and balance (30, 31). Studies have shown that as compared to CE, Taichi can significantly improve the cognitive performance in older adults. For example, Lam and colleagues observed that compared to CE consisting of stretching and toning, one-year training of Taichi can induce greater improvement in the performance of delay-recall task and better preservation of Clinical Dementia Rating scores in a group of older adults (32). This may thus be another important aspect pertaining to the observed benefits of Tachi for improvement in functional mobility and balance (33, 34). More studies are needed to more explicitly explore the potential pathways underneath the benefits of Taichi for functional mobility and balance in older adults.

The protocol design of Taichi is critical to its effectiveness on functional mobility and balance. Regarding to the style of Taichi, we showed that Yang style is more appropriate than Sun style. This is consistent with a previous meta-analysis showing greater effects of Yang style on the reduction of falls as compared to Sun style (35). By looking into the protocol of these two types of Taichi, Sun style consists of high stance, narrow feet distance, fast motion switching, and multiple follow-up steps (20); while Yang style consists of slow, large, graceful, sequential movements from one pose to the next with an upright posture and high stance position (36), thus practicing balance control more. However, no study to date has directly compared the effects of these two styles on balance and functional mobility. It is thus worthwhile to examine the appropriate style for the capacity of balance control in future studies. More interestingly, we here provide evidence that Taichi within shorter intervention length or total time can induce greater improvements compared to CE, indicating Taichi would be a more efficient intervention by simultaneously augmenting multiple underlying functions that are critical to functional mobility and balance. It was observed in previous studies that greater effects of Taichi on fall prevention and static balance were associated with higher frequency and greater session number of Taichi (35, 37). It is thus highly demanded to more explicitly examine the association between the number and frequency of sessions, as well as the length of each session of Taichi, and its effects on functional mobility and balance (i.e., “dose–response” relationship) in older adult populations, the knowledge obtained from which will ultimately help the design of Taichi intervention to maximize its benefits.

Several limitations should be noted. Only 12 publications were included in this work, potentially limiting the power of the evidence. Due to limited information provided in these publications, we did not perform subgroup analyses for other aspects (e.g., the effects of Chen style, which was reported in only one study). This suggests that more work is needed in this field to further examine and confirm the current findings. The publication bias resulted in heterogeneity between included studies. The participant characteristics were still different across studies, though increasing the generalizability of observation, the effects of Taichi on different populations should be carefully assessed and confirmed in future.

## 5 Conclusion

Our work suggests that in relatively healthy older adults, when the exercise duration is limited, Taichi, especially Yang style, would be more beneficial for their functional mobility and balance as compared to CE, which is worthwhile to be taken into consideration in the design of future rehabilitation programs.

## Data availability statement

The original contributions presented in the study are included in the article/Supplementary material, further inquiries can be directed to the corresponding author.

## Author contributions

YL: Writing – original draft, Writing – review & editing. ML: Writing – review & editing. KZ: Writing – review & editing. GD: Writing – review & editing. BM: Writing – review & editing. DB: Writing – review & editing. JZ: Writing – review & editing.
