# Supplementary material for: The comparison between effects of Taichi and conventional exercise on functional mobility and balance in healthy older adults: a systematic literature review and meta-analysis
Source: Front Public Health. 2023 Dec 18;11:1281144. doi: 10.3389/fpubh.2023.1281144 (PMC10757983; doi:10.3389/fpubh.2023.1281144)
Supplement: Supplementary file 1 [file Table_5.DOCX]

Supplementary Material

# Supplementary Text

## S1 Search Strategy

| #1 | Tai Ji OR Tai-ji OR Tai Chi OR Taichi OR Tai Ji Quan OR Taiji OR Taijiquan OR T'ai Chi OR Tai Chi Chuan OR traditional Chinese exercise OR traditional Chinese health exercise OR traditional Chinese mind and body exercise |
| --- | --- |
| #2 | functional mobility OR Functional movement OR Functional status OR Functional Independence OR Functional Dependence OR Physical Functional performance OR Physical Performance OR locomotion OR Locomotor Activit* walk OR walking OR balance OR postural balance OR motor activit* OR Ambulation OR Posture Equilibrium* OR Posture Balance* OR Musculoskeletal Equilibrium OR Postur* Control* OR Timed-Up-and-Go OR timed up and go OR TUG OR sit to stand |
| #3 | aged OR Elderly OR older adults OR older people OR senior OR oldest old OR aged patient* OR aged individual OR oldest adults OR old patients OR older veterans OR older men OR older women OR geriatrics OR age 60 OR age 65 OR age 70 OR age 75 OR age 80 |
| #4 | randomized controlled trial OR randomized OR placebo OR RCT |
| #5 | #1 AND #2 AND #3 AND #4 AND (Publication date: 19000101-20230131) |

# Supplementary Figures

## Figure S1 Risk of bias graph


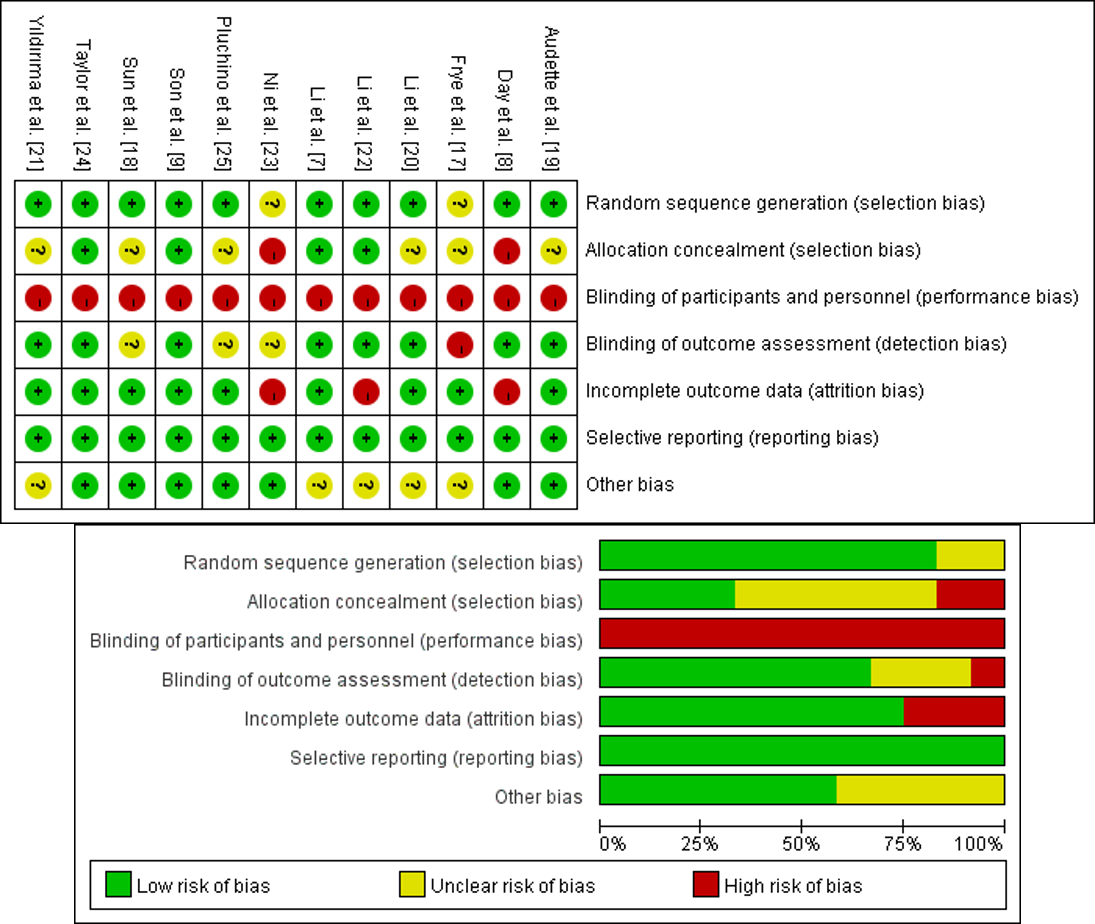


## Figure S2 Forest plots of publication bias


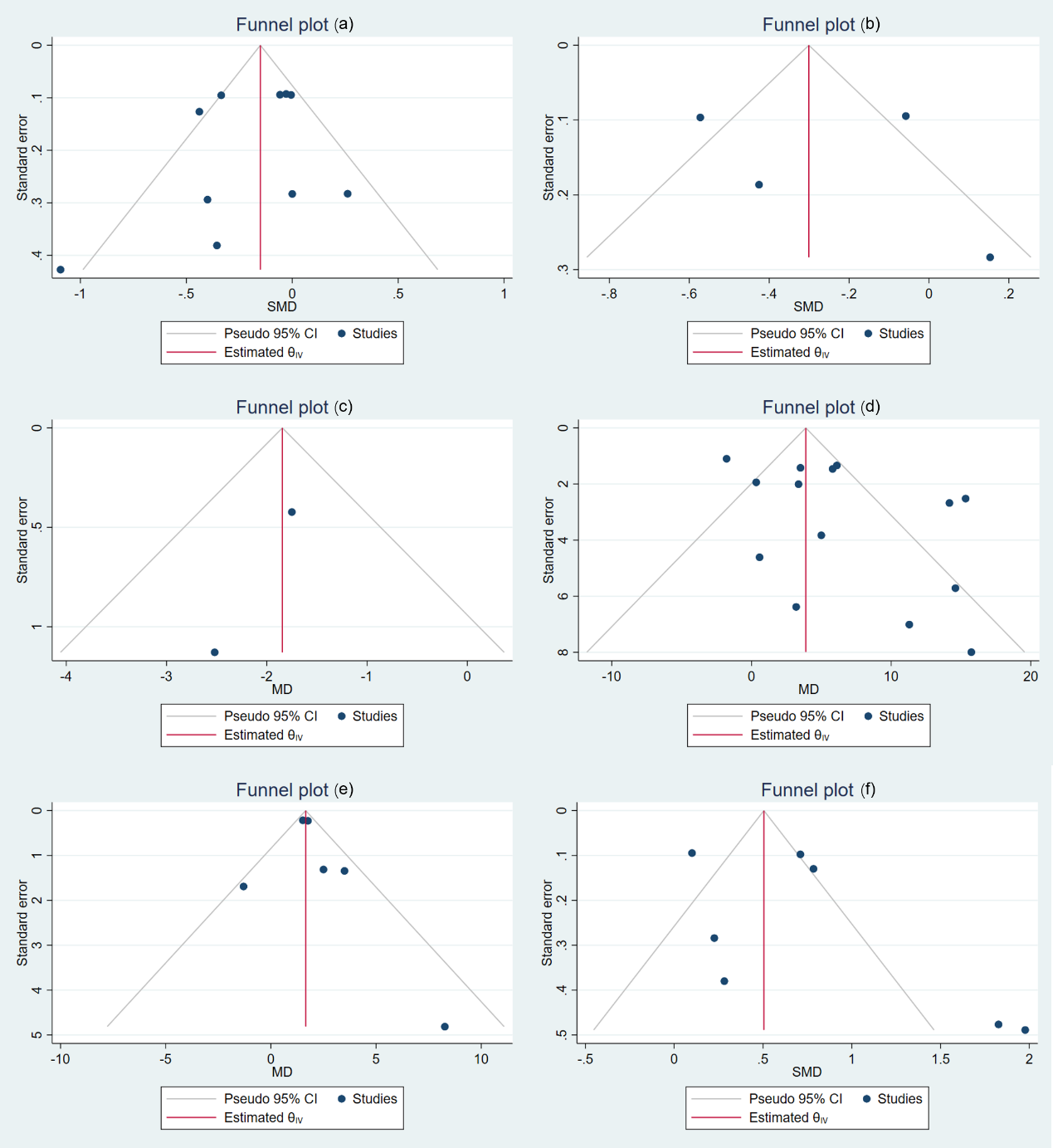


Funnel plots for all meta-analysis: (a)TUG; (b) STS; (c) 50-foot walk; (d)OLS-O; (e)OLS-C; (f) FR
